# Brain metastasis from colorectal cancer: Treatment, survival, and prognosis

**DOI:** 10.1097/MD.0000000000030273

**Published:** 2022-10-07

**Authors:** Wenxia Li, Tongsheng Wang, Yubing Zhu, Haijiao Yu, Ling Ma, Yuhan Ding, Gao Hong, Ding Lei

**Affiliations:** a Department of Oncology Surgery, Beijing Shijitan Hospital, Capital Medical University, Peking University Ninth School of Clinical Medicine, Beijing, China.

**Keywords:** brain metastasis, colorectal cancer, prognosis, survival, treatment

## Abstract

To investigate the clinical characteristics, survival, prognostic factors, and treatment of brain metastasis (BM) from colorectal cancer (CRC). Twenty-one patients with BM from CRC were retrospectively reviewed. Predictive factors for BM and prognostic factors after the diagnosis of BM were examined by univariate and multivariate COX analysis. The time from the development of extracranial metastases, including lung, bone, and liver, to the occurrence of BM was recorded separately. The median overall survival time was 7 months. In univariate prognostic analysis, median survival with multimodal therapy was better than that with unimodal therapy (10 months vs 3 months, *P* = .000). In addition, median survival with Karnofsky performance status (KPS) < 70, 1 BM lesion, primary tumor stage of II-III, extracranial lesions < 2, and no extracranial metastasis were much better than the other groups (*P* < .05 of all). Although there was not a significant difference in median survival between patients receiving combination treatment with bevacizumab and those who did not, treatment with bevacizumab was associated with better survival (10 months vs 5 months, *P* = .436). The time intervals from bone, liver, and lung metastases to BM were 3, 6.5, and 11 months, respectively. Based on multivariate Cox analysis, KPS and treatment modalities were independent prognosis factors (*P* = .039 and *P* = .000, respectively). CRC patients with a high KPS and multimodal treatment have improved survival.

## 1. Introduction

Colorectal cancer (CRC) is one of the most common malignancies and the leading cause of cancer-related death.^[1–3]^ To the best of our knowledge, recurrence and metastasis are the primary causes of death in patients with CRC. The liver, lungs, and peritoneal cavity are the most common metastatic sites in patients with CRC, while brain metastasis (BM) is relatively rare. BM is a common complication of lung cancer (40%–50% of cases), testicular cancer (10%–15%), breast cancer (5%–15%), and melanoma (10%).^[3,4]^ Clinical guidelines rarely recommend screening for BM in patients with CRC; the crude incidence of BM from CRC is 0.27%^[4]^ but with improved diagnostics and treatment techniques, the reported incidence increases to 9%.^[5–9]^

To the best of our knowledge, the Karnofsky performance status (KPS) score is recommended for assessing the prognosis and treatment outcomes of CRC. As an important variable in the diagnosis-specific graded prognostic assessment, the KPS has been shown to be an independent adverse prognostic factor for BM in CRC,^[10–12]^ although that finding is controversial.^[13]^

Many studies suggest that extracranial metastasis may occur earlier than BM. Christensen et al^[7]^ found that lung metastases at the time of metastatic colorectal cancer diagnosis and the presence of rectal cancer significantly increased the risk of developing BM according to both univariate and multivariate Cox regression analyses. In recent years, researchers have begun to pay attention to the relationship between gene mutations, especially rat sarcoma viral oncogene homolog (RAS) gene mutations and BM from CRC. Zheng Hu et al^[14]^ indicated that mutations in the canonical “core” genes (Kirsten rat sarcoma viral oncogene homolog [KRAS], Adenomatous polyposis coli, etc) are often observed in patients with BM from CRC. Similar observations were described by other authors.^[5,15]^ However, contrasting observations have also been reported.^[7,12]^ Research on the relationship between RAS mutations and prognosis is limited.

The survival time of patients with BM is quite poor, with a median survival of only 1 to 2 months without treatment.^[3,16]^ In recent years, local treatments, such as several types of radiation (whole-brain radiotherapy [WBRT] and stereotactic radiosurgery), and neurosurgical resection have been widely applied to metastatic lesions.^[5,13,16–19]^ Previous research has shown that the blood–brain barrier blocks the passage of cytotoxic drugs, so the effect of chemotherapy is poor. Recent studies have shown that advances in multimodal treatments such as combinations of radiotherapy, chemotherapy, and targeted therapies have improved the prognosis of patients with unresectable CRC, with a median overall survival (OS) of 4 to 12 months.^[8,10,20]^ The OS of patients with BM from CRC is extremely poor.

Although increasing number of studies have been performed regarding BM in CRC, the data are still limited due to the relative low incidence. The aim of this study was to examine the biological and clinical characteristics at the initial presentation that could predict the later development of BM, evaluate the prognosis of patients with BM from CRC, and estimate the outcomes of several types of therapy for BM from CRC.

## 2. Methods

### 2.1. Patients

A retrospective analysis of BM from CRC was performed. Clinical data were collected from patients seen between January 2012 and February 2020 at Beijing Shijitan Hospital Affiliated with Capital Medical University (Beijing, China). The 21 patients were diagnosed with CRC based on pathology and BM based on computed tomography or magnetic resonance imaging; patients with BM who underwent neurosurgery also received a pathologically proven diagnosis. In total, 20/21 patients were diagnosed with colorectal adenocarcinoma, and 1 patient had sigmoid colon signet ring cell carcinoma that progressed with a secondary meningeal malignancy. This study was approved by the Ethics Committee of Beijing Shijitan Hospital Affiliated with Capital Medical University. All the patients signed the informed consent.

### 2.2. Data collection

The patients’ medical records were reviewed, including sex; age at BM diagnosis (≤60, >60 years) KPS (<70, ≥70); KRAS exon 2 status (wild type, mutated); number of BM lesions (1, ≥2); TNM stage of the primary tumor (II-III, IV); primary tumor location (left hemicolon and rectum; right hemicolon); extracranial lesions (<2, ≥2); extracranial metastasis (yes, no); treatment modalities for BM (unimodal treatment, multimodal treatment); combination therapy with bevacizumab (yes, no); neurological symptoms; and survival time.

Single treatment modalities of BM were categorized as WBRT alone; and traditional chemotherapy alone (including oxaliplatin or irinotecan); multimodal treatment was categorized as chemotherapy + neurotherapy + radiotherapy; chemotherapy + neurosurgery/radiotherapy/immunotherapy; chemotherapy + bevacizumab/cetuximab. The time from the appearance of extracranial metastases to the development of BM was recorded.

### 2.3. Statistical analysis

The statistical analyses were performed with IBM SPSS Statistics version 25.0 (IBM Co., Armonk, NY). Clinicopathological data are presented as the medians and ranges for continuous variables. The independent prognostic factors were estimated using multivariate analyses with Cox proportional hazards models. Median survival times were analyzed using the Kaplan–Meier method and groups were compared using the log-rank test. *P* values <.05 were regarded as statistically significant.

## 3. Results

### 3.1. Overall analyses

A total of 21 patients with BM from CRC were enrolled in this retrospective study. Table 1 summarizes the clinical and tumor characteristics of all included patients. The majority of patients with BM were male (12 patients, 57%). The age at the development of BM ranged from 19 to 76 years. Eight patients had poor KPS (KPS < 70). The left hemicolon and rectum were the most common primary locations for CRC that metastasized to the brain (16 patients, 76%). There were 8 patients with KRAS exon 2 mutations and 7 with wild-type KRAS exon 2; another 6 patients had unknown KRAS exon 2 statuses. The majority of patients (17, 81%) presented with extracranial metastases and the lung was the most common site of extracranial metastasis (11 patients). Almost all the patients with BM had neurological symptoms, including headache, dizziness, nausea, vomiting, blurred vision, disturbance of consciousness, motor disturbance, speech difficulty, and memory impairment. Among these symptoms, motor disturbance was the most common, followed by headache/dizziness.

**Table 1:** Clinicopathologic features of patients and univariate prognostic analysis of patients with BM from CRC.

Variables	Patient no.	Median OS (months)	95% CI	*P* value
Overall	21	7	2.514–11.486	
Sex				.989
Male	12	6	0.000–12.790	
Female	9	7	1.156–12.844	
Age at the BM (yr)				
≤60	11	6	0.000–13.445	.951
>60	10	7	2.352–11.649	
KPS				
<70	8	4	1.367–6.633	.039
≥70	13	10	2.954–17.046	
KRAS exon 2 status				
Wild type	7	5	2.434–7.566	.587
Mutated type	8	8	5.228–10.772	
NA	6	4	1.199–6.801	
Number of BM lesions				
1	7	11	8.434–13.566	.000
≥2	12	5	1.605–8.395	
NA	2			
Primary tumor stage				.047
II–III	14	8	0.666–15.334	
IV	7	4	2.845–5.155	
Primary tumor location				.085
Left hemicolon and rectum	16	9	1.160–16.840	
Right hemicolon	5	4	2.068–5.932	
Extracranial lesions at the BM				.017
<2	14	10	2.666–17.334	
≥2	7	5	0.000–10.132	
Extracranial metastasis at the time of the diagnosis of BM				.040
Yes	17	5	2.311–7.689	
No	4	12	0.000–30.620	
Treatment modalities after BM				.000
Unimodal treatment	5	3	2.463–3.537	
Multimodal treatment	16	10	6.111–13.889	
Combination with bevacizumab				.436
Yes	6	10	6.339–13.601	
No	15	5	3.234–6.766	
Neurological symptoms				
Headache/dizziness	10			
Nausea/vomiting	3			
Blurred vision	3			
Motor disturbance	13			
Speech difficulty	4			
Memory impairment	1			
Asymptomatic	1			
Disturbance of consciousness	3			

BM = brain metastasis, CI = confidence interval, CRC = colorectal cancer, KPS = Karnofsky performance status, KRAS = Kirsten rat sarcoma viral oncogene homolog, OS = overall survival.

### 3.2. Treatment modalities

In terms of treatment, unimodal treatment was administered to 5 patients. Most patients received multimodal treatment, and the details were as follows: 3 patients received chemotherapy and neurosurgery/radiotherapy; 5 patients received both chemotherapy and radiotherapy or neurotherapy or immunotherapy; and 8 patients received chemotherapy and targeted therapy; 6 of whom received bevacizumab; and 2 who received cetuximab (Table 2).

**Table 2:** Time interval from the occurrence of extracranial metastasis to the development of BM.

Time	Patients no.	Median time interval months (range)
Time interval from the occurrence of bone metastasis to the development of BM	7	3 (0–13)
Time interval from the occurrence of liver metastasis to the development of BM	6	6.5 (0–12)
Time interval from the occurrence of lung metastasis to the development of BM	11	11 (0–29)

BM = brain metastasis.

### 3.3. Time to brain metastasis

The median time interval from the diagnosis of CRC to the development of BM was 18 months. A diagnosis of extracranial metastases significantly increased the risk of developing BM from CRC. As shown in Table 3, the time interval from the occurrence of extracranial metastases to the development of BM was recorded and analyzed. The median time from the occurrence of lung metastasis to the development of BM was 11 months (range 0–29 months), the time from liver metastasis to BM was 6.5 months (range 0–12 months), and the time from bone metastasis to BM was 3 months (range 0–13 months).

**Table 3:** Treatment modalities for brain metastasis.

Treatment modality	Patients no. (n)	Percentage
Unimodal treatment		
Chemotherapy only	2	10
Radiotherapy only	3	14
Multimodal treatment		
Chemotherapy + Neurosurgery + Radiotherapy	3	14
Chemotherapy + Radiotherapy/Neurotherapy/Immunotherapy	5	24
Chemotherapy + Bevacizumab	6	28
Chemotherapy + Cetuximab	2	10

### 3.4. Survival and prognostic factors after the development of brain metastasis

Overall, 2 patients survived. The median OS was 7 months (95% confidence interval (CI), 2.514–11.486 months), and the 1-year and 2-year OS rates were 19.05% and 9.52%, respectively.

**Figure 1.**
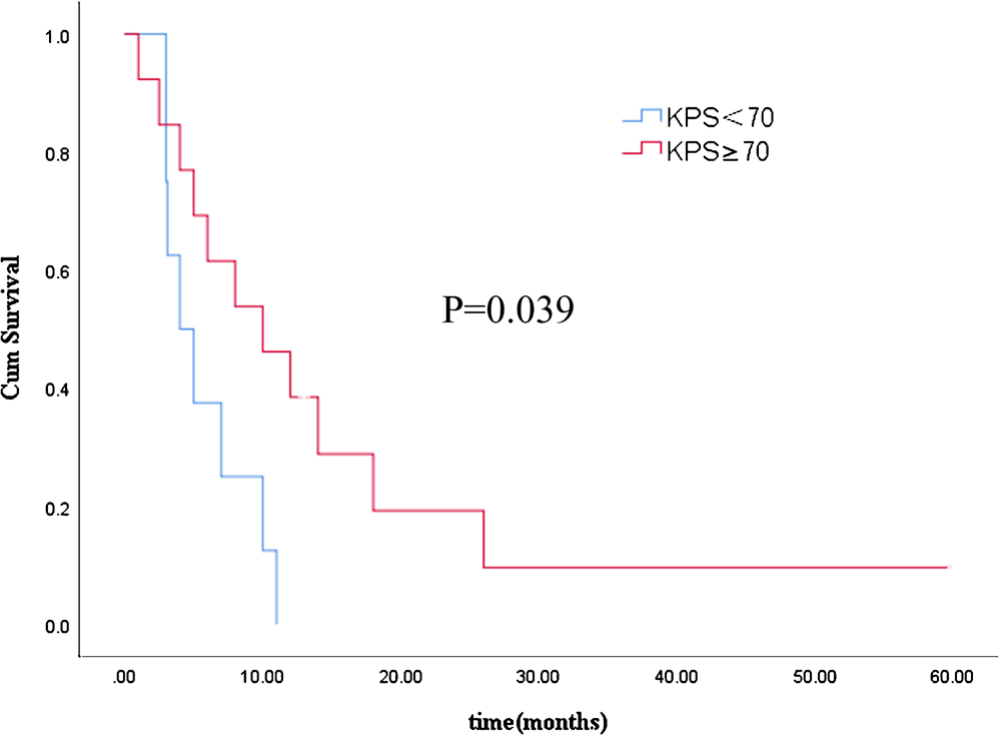
Kaplan–Meier survival analysis of patients with different KPS. KPS = Karnofsky performance status.

**Figure 2.**
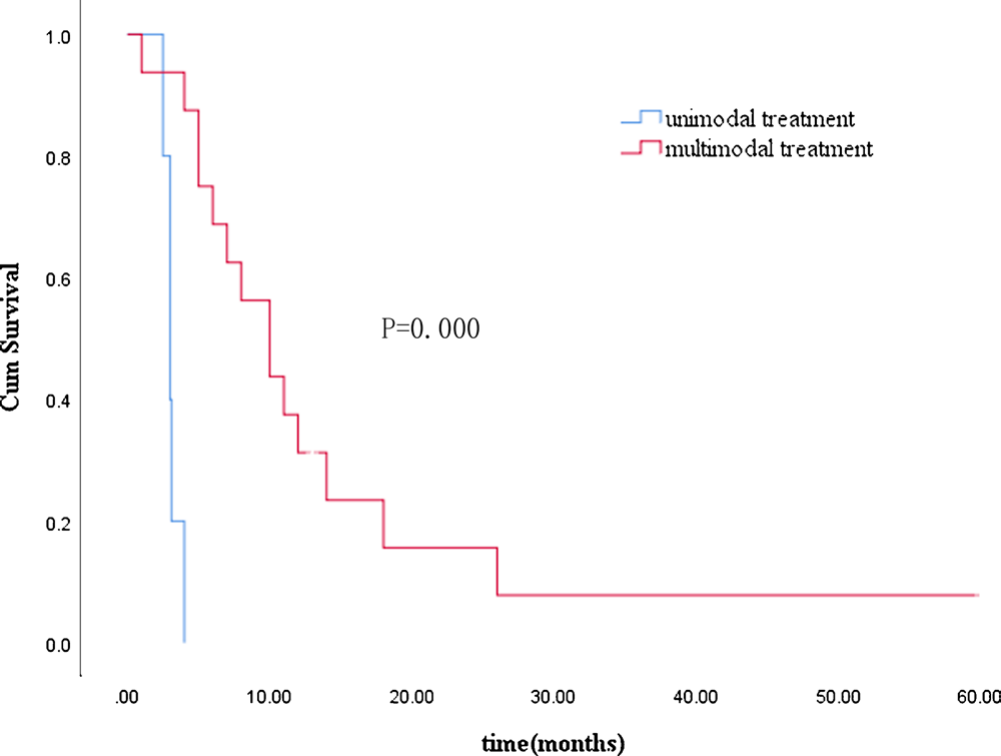
Kaplan–Meier survival analysis of patients with unimodal and multimodal treatment.

The results of univariate analyses of median OS from the time of the diagnosis of BM are shown in Table 1. Univariate analysis showed that the number of BM lesions, primary tumor stage, the presence of extracranial lesions at the time of the diagnosis of BM, and treatment modalities for BM were significant prognostic factors. There was no significant difference in survival according to: sex, age, KRAS 2 exon mutation status, primary tumor location, or administration of bevacizumab. The variables found to be significant in univariate analysis were included in multivariate analysis, and we found that the KPS (hazard ratio 0.211, 95% confidence interval (CI) 0.047–0.956, *P* = .044) and treatment modalities for BM (hazard ratio 0.063, 95% CI 0.013–0.689, *P* = .024) were independent prognostic factors (Table 4). The median survival time of patients with a KPS ≥ 70 (10 months, 95% CI 2.954–17.046 months) was better than that of patients with a KPS < 70 (4 months, 95% CI 1.367–6.633 months) (*P* = .039). The median survival time of patients who received multimodal treatment (10 months, 95% CI 3.234–6.766 months) was better than that of patients who received unimodal treatment (3 months, 95% CI 2.463–3.537 months) (*P* = .000). The Kaplan–Meier curves for the KPS and treatment modalities are shown in Figures 1 and 2.

**Table 4:** Significant factors affecting the prognosis in multivariate analysis.

Variables	HR	95% CI	*P* value
KPS			.044
<70	1	0.047–0.956	
≥70	0.211		
Treatment modalities after BM			.024
Unimodal treatment	1		
Multimodal treatment	0.063	0.013–0.689	

BM = brain metastasis, CI = confidence interval, HR = hazard ratio, KPS = Karnofsky performance status, HR = hazard ratio.

**Figure 3.**
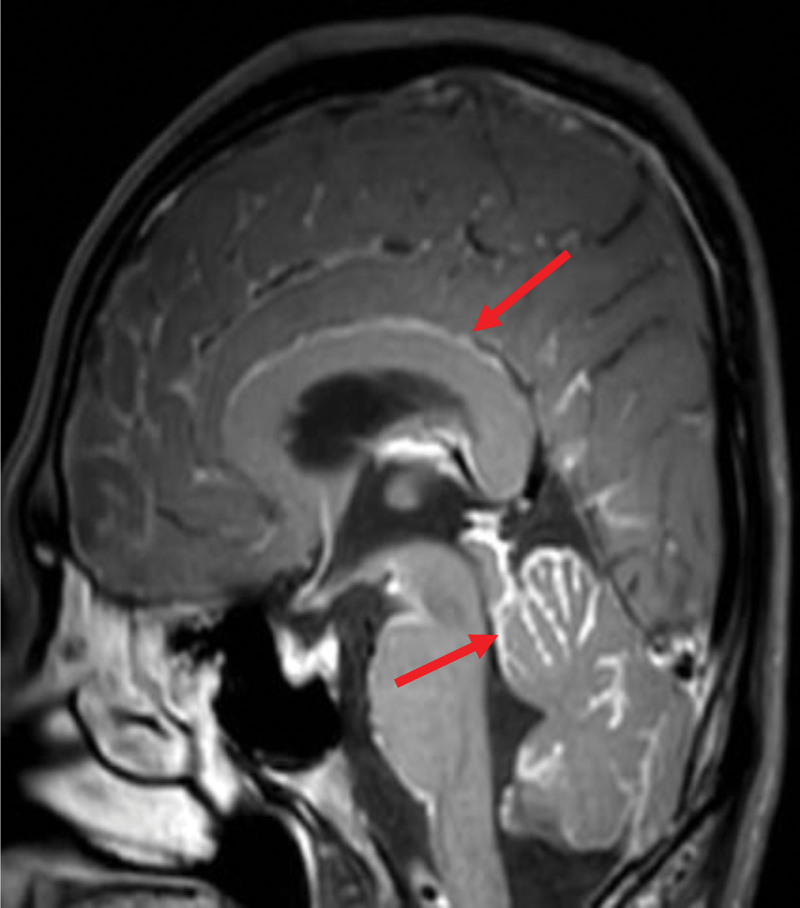
Magnetic resonance of BM with meningioma. BM = brain metastasis.

One patient had pathologically confirmed signet ring cell carcinoma of the sigmoid colon, BM was diagnosed 3 months after surgery, and the BM was a meningioma (Fig. 3). The OS time was 1 month after the diagnosis of BM.

## 4. Discussion

Metastasis to the brain is associated with extremely poor outcomes. BM from CRC is uncommon, and clinical guidelines such as those from the National Comprehensive Cancer Network rarely recommend routine screening for BM in patients with CRC, making it easy to miss in the clinical setting.

Overall, the prognosis for patients with BM from CRC is generally regarded as poor, and the median survival time is only a few months.^[11,16,21,22]^ In this study, the median survival time was 7 months. Several studies found that the reliable factors predictive of a poor prognosis were recursive partitioning analysis class III,^[18,23]^ KPS < 70,^[10,11]^ diagnosis-specific graded prognostic assessment score: 0 to 1,^[19]^ extracranial metastases,^[13,24,25]^ treatment,^[12,20,26,27]^ number of BMs, and the specific targeted therapy agents.^[13,26]^ However, there were differences among studies because of selection bias and small numbers of patient. The KPS, number of BM lesions, primary tumor stage, extracranial lesions at the time of the diagnosis of BM, and treatment modalities for BM were identified as independent prognostic factors in univariate prognostic analysis. In multivariate analysis, KPS ≥ 70 and multimodal treatment predicted better survival.

KPS is based on physicians’ clinical estimation and were used to assess patients’ functional status. As we know, patients who suffered malignant tumor with better physical status have satisfactory prognosis. Lu et al^[10]^ concluded that KPS was an independent prognostic factor for patients with BM from CRC, and patient with KPS > 70 could provide an additional survival benefit. Similar conclusions were also found.^[28]^ In our study, we observed that patients with better physical status (KPS ≥ 70) can get better survival improvements than patients with poor physical status (KPS < 70) (10 months vs 4 months), both in univariate analysis and in multivariate analysis.

The majority of studies found that RAS mutations were independently associated with the development of BM,^[15,29]^ although reports on the association of RAS mutations with the prognosis of BM from CRC have been limited. Fountzilas et al^[23]^ collected data from 40 patients with BM from CRC and showed that activating KRAS mutations correlated with OS but not after the development of brain lesions, which was consistent with the conclusion drawn in this study. Our results indicate that patients with wild type of KRAS have an unsatisfactory survival; however, there was no statistical difference. In the future, studies with large sample sizes are needed to further support the correlation between RAS status and survival of patients with BM from CRC.

Similar to previous reports,^[3,5,11]^ our results showed that BM from CRC tended to occur in patients who had extracranial metastases. In our analysis of extracranial metastasis, lung metastasis was more frequent than metastasis to liver and bone. These clinical characteristics occur maybe due to the differences in venous drainage from the colorectum.^[18]^ Thompson et al^[30]^ performed a retrospective analysis of patients with BM from CRC, and they found that bone, lung, and distant node metastasis were independent predictors of BM on multivariate logistics regression. Quan et al^[11]^ analyzed BM in 52 patients with CRC and concluded that the lung (59.6%) was the most common extracranial metastasis location, followed by the liver (36.5%) and bone (21.2%). The results were consistent with those in the study by Xiao-Dong Gu et al.^[13]^ Therefore, monitoring extracranial metastasis after CRC was benefit to BM. Unfortunately, there are no guidelines regarding the follow-up period. In this study, we analyzed the time between the occurrence of the above 3 types of extracranial metastases and the development of BM. By calculating these time intervals, we found that the median time from bone metastasis to BM was only 3 months, the time from liver metastasis to BM was 6.5 months, and the time from lung metastasis to BM was 11 months. By defining the time and risk factors from the occurrence of extracranial metastases to the development of BM, we can detect the occurrence of BM and also establish reasonable follow-up schemes to enable the earlier detection of BM from CRC.

The blood–brain barrier is a physical barrier. The blood–brain barrier is adversely affected by BM from CRC due to the loosening of the tight junctions between endothelial cells. However, despite the increased permeability of the brain parenchyma, most systemic chemotherapeutic agents are still too large to cross the blood–brain barrier.^[31]^ Current investigators have demonstrated that BM occurs when tumor cells enter the brain.^[18]^ Previous treatment of BM, typically including neurosurgery, radiotherapy, or systemic medical therapies, has not been as effective as clinicians expected.^[32,33]^ Besides, the standard treatment for BM of CRC has not yet been established. Currently, multimodal treatment including surgery, radiotherapy, chemotherapy, and targeted therapy has been accepted for metastases from CRC, including BM. Xingang Lu and colleagues analyzed 80 patients with BM from CRC and observed that the median OS in patients with unimodal treatment was only 4 months; however, multimodal treatment combining neurosurgery with radiotherapy (WBRT or stereotactic radiosurgery) or chemotherapy resulted in a better survival time (median OS 11 months).^[10]^ Dong-Yeop Kim et al^[18]^ drew a similar conclusion. We also found that patients receiving multimodal treatment survived longer than those receiving unimodal treatment (10 months vs 3 months). In recent years, targeted therapy has been included in the treatment of BM from CRC. Considering the occurrence of intracerebral bleeding in a pharmacokinetic trial, patients with BM were not treated with bevacizumab in the past.^[34,35]^ Nevertheless, in daily clinical practice, no increased risk of intracerebral bleeding has been reported in patients treated with bevacizumab. Fabian Finkelmeier et al^[20]^ collected data from 5 patients with BM from CRC treated with a bevacizumab chemotherapy regimen following either neurosurgery, radiosurgery, or WBRT. The OS from the diagnosis of BM was 20.6 months (7–42 months) and no intracranial hemorrhage was observed. In this study, we observed that the survival of patients treated with bevacizumab was better than that of those not treated with bevacizumab, although the difference was not significant. The lack of significance may be due to the small sample size. In addition, temozolomide is a new alkylating agent that can cross the blood–brain barrier and sensitize malignant cells to radiotherapy. A study from China showed that treatment with temozolomide combined with WBRT was effective and safe compared to WBRT alone. A case report of a woman with multiple liver, lung, and brain metastases indicated that the third-line regimen of combined TFTD and bevacizumab therapy after radiotherapy significantly prolonged her OS time to 1 year and was effective at reducing brain edema.^[36]^ In the future, more studies on new therapeutic agents are needed to improve the survival of patients with BM from CRC.

Our study is limited by several factors. First, it was a single-center study, and because of low incidence, the sample size was small. The risk factors of BM were insufficient, therefore, identifying the high-risk factors with BM from CRC is inadequate, and further establishing the effect follow-up scheme is absolutely necessary. Second, this was a retrospective study, so the data were incomplete, and selection bias was inevitable. Third, the study lasted for a little long time, and some treatments (targeted therapy, etc) may have been widely used only in recent years, so the early treatment is mainly single treatment, which will slightly affect the research results. In the future, more large-scale cohort studies are needed to better estimate the survival and prognosis of patients with BM from CRC.

In conclusion, CRC metastatic to the brain is a highly lethal condition. We herein demonstrated that the KPS and treatment modalities after BM were independent prognostic factors. Additionally, we identified the time from extracranial metastases to BM, which may be conducive to the development of follow-up strategies in future. In clinical practice, once patients with CRC have extracranial metastasis, it is necessary to closely observe neurological symptoms in order to detect BM early. Since the prognosis remains poor, it is important to determine the optimal treatment regimen based on the characteristics of the available therapeutic options.

## Author contributions

**Data curation:** Yubing Zhu, Haijiao Yu.

Formal analysis: Ling Ma.

Methodology: Yuhan Ding.

Supervision: Tongsheng Wang.

Writing – original draft: Wenxia Li.

Writing – review & editing: Gao Hong, Ding Lei.
